# Three-dimensional reconstruction of ediacaran ceramiales (*Rhodophyta*) from the phosphorite doushantuo formation, South China

**DOI:** 10.1038/s41598-026-42410-5

**Published:** 2026-03-06

**Authors:** Wei Du, Xunlian Wang, Yue Wang, Kentaro Uesugi, Masahiro Yasutake, Tsuyoshi Komiya

**Affiliations:** 1 Graduate School of Arts and Sciences, The University of Tokyo, Tokyo, 153-8902 Japan; 2 School of Earth Sciences and Resources, China University of Geosciences, Beijing, 100083 China; 3 School of Resource and Environment, Guizhou University, Guiyang, 550003 China; 4 Japan Synchrotron Radiation Research Institute (JASRI), 1-1-1 Kouto, Sayo-Cho, Sayo-Gun, Hyogo 679-5198 Japan

**Keywords:** Ceramiales, Rhodophyta, Ediacaran, Doushantuo formation, Cell biology, Evolution

## Abstract

The Ediacaran Doushantuo Formation (551–635 Ma) is known to include excellently preserved microfossils that reveal information about early eukaryotic evolution. Here, we report the presence of Vetusceramium sinense gen. et sp. nov., a new multicellular red algal fossil from the Weng’an Phosphorite Member of the Doushantuo Formation, South China. Reconstruction of the fossils using synchrotron X-ray tomography shows that these specimens bear a multilayered uniaxial thallus comprising a large central axial cell, pericentral corticated cell filaments, and possible pit connections, which bear similarity to the branches of modern Ceramiales belonging to Rhodophyta. However, the absence of reproductive features, such as gametangia, prevents crown group classification, suggesting a stem group affinity within the Ceramiales. This evidence suggests the possibility of pushing back the occurrence of Ceramiales-like multicellularity to the late Ediacaran Period, demonstrating sophisticated developmental strategies in early photoautotrophs. This report highlights the importance of phosphatization in uncovering subcellular details and offers key insights into the evolution of red algae before the Cambrian radiation.

**Supplementary Information:**

The online version contains supplementary material available at 10.1038/s41598-026-42410-5.

## Introduction

Reconstructing the early evolution of red macroalgae (Rhodophyta) during the Precambrian is significantly hindered by inherent preservation biases. The soft-bodied nature of most taxa, combined with the frequent absence of diagnostic features, such as reproductive structures or biochemical signatures in the fossil record, complicates taxonomic assignment and phylogenetic interpretations^1^. Although a few Proterozoic fossils provide evidence for early rhodophyte diversity, their interpretations vary. *Bangiomorpha pubescens* (~ 1.05 Ga) is widely accepted as a member of the red algal total group^2^, whereas the oldest record of red algae, ~ 1.6 Ga *Ramathallus*, was reported from the Vindhyan Supergroup of central India^3^. However, its Rhodophyte and Archaeplastida interpretations are controversial^2,4,5^. Younger Ediacaran fossils from the Doushantuo Formation (~ 0.6 Ga), including forms interpreted as Florideophytes^6–9^, often lack definitive algal morphologies, such as pit connections, a key synapomorphy of Florideophyceae, and photosynthetic structures, leading to systematic uncertainties and provisional taxonomic placement^1,10^. The complex pseudoparenchymatous thalli of *Thallophyca* and *Paramecia* have been interpreted as stem group corallinaceans^11^, yet they differ from crown group corallines in that they are not calcified during their lifetime^5,12,13^. Molecular clock analyses place the divergence of major rhodophyte lineages deep within the Proterozoic (e.g., Florideophyceae ~ 800 Ma)^14,15^ and suggest that the highly diverse order Ceramiales diverged later in the Paleozoic (~ 335–400 Ma)^16,17^. However, the pre-Paleozoic fossil record of the Ceramiales is exceptionally sparse. Definitive crown group Ceramiales fossils are unknown from the Precambrian, and even younger putative examples, such as the Eocene *Pterigophycos*, possess unresolved systematic affinities^18^. This gap obscures the origins and early evolution of this major order of algae.

Here, we describe a new macroalga, *Vetusceramium sinense* gen. et sp. nov., from the Ediacaran Doushantuo Formation (~ 551–635 Ma)^10,19^. This fossil exhibits a multilayered uniaxial thallus characterized by large central axial cells, corticated pericentral filaments, and structures potentially analogous to the pit connections of extant Ceramiales. Although the absence of critical traits, such as gametangia, necessitates cautious phylogenetic placement, *V. sinense* provides significant new morphological data pertinent to the understanding of late Proterozoic Florideophyceae diversification. Phylomorphospace analysis suggests a stem group affinity relative to crown Ceramiales, a finding consistent with its Ediacaran age but highlighting the significant temporal gap preceding the estimated crown-group divergence.

## Geological setting and fossil preservation

### Geological setting

**Fig. 1:**
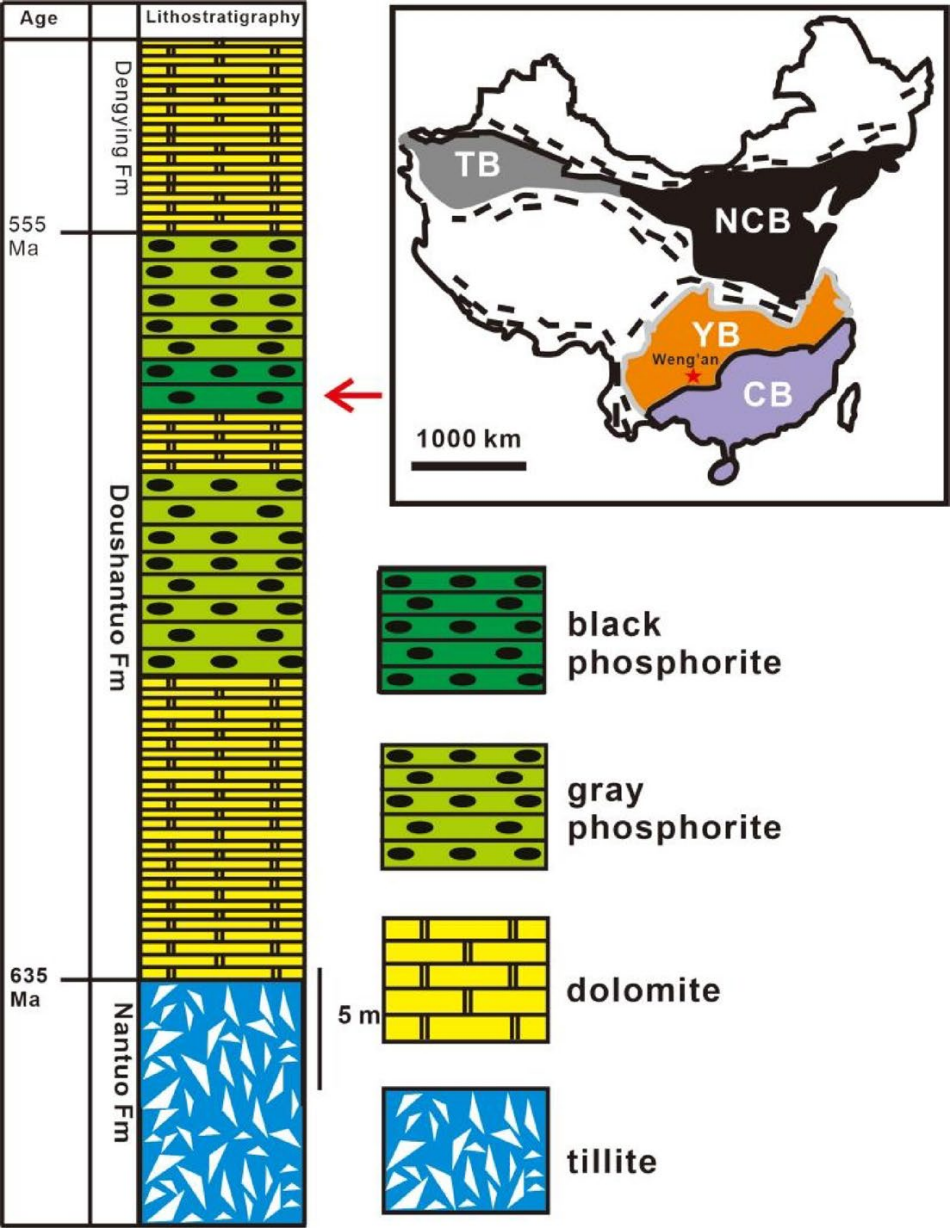
Geotectonic map of China and stratigraphic column of the fossiliferous Mt. Beidoushan section in Weng’an, Guizhou Province. The geotectonic map shows the relationships between the Yangtze Block (YB), North China Block (NCB), Tarim Block (TB) and Cathaysia Block (CB). A red star marks the location of the Beidoushan section in Weng’an area. The red arrow in the stratigraphic column indicates the fossiliferous layer. Fm, Formation.

All illustrated fossils originate from the black bituminous facies of the embryo-bearing Weng’an Phosphorite Member of the Ediacaran Doushantuo Formation (551–635 Ma)^19^. This locality, situated near Weng’an City in Guizhou Province, South China (Fig. 1), is globally significant for its exceptional preservation of spherical microfossil assemblages, which have been interpreted as potential animal embryos, algal structures, and early metazoans^9^. The Doushantuo Formation unconformably overlies the Marinoan glacial diamictites of the Cryogenian Nantuo Formation (~ 635 Ma) and is overlain by the Dengying Formation, which hosts classical Ediacaran macrofossils^6,20–23^.

The Weng’an Phosphorite Member comprises two distinct lithofacies: lower black bituminous phosphorite and upper gray phosphorite^9,24,25^. Petrographic analyses reveal that the black facies experienced minimal post-depositional reworking and contained elevated organic matter, creating a broader taphonomic window for preserving delicate biological structures^24^. Although previous studies prioritized gray facies because of easier fossil extraction, this study focuses on the understudied black facies, where phosphate impregnation of cellular structures and rapid authigenic mineralization inhibited decay, preserving subcellular details^9,25^.

### Fossil preservation mechanisms

The exceptional preservation of the Doushantuo microfossils is attributed to their deposition in shallow, oxygenated marine environments with high phosphate saturation^23,26^. Rapid phosphatization stabilized soft tissues and cellular structures, while organic-rich sediments in the black facies further inhibited microbial degradation. Fossilized structures often exhibit phosphate-encrusted cuticles and intracellular mineral infills, preserving fine anatomical details, such as embryonic cells and putative cleavage stages^23^.

## Result

### Fossil description

**Fig. 2:**
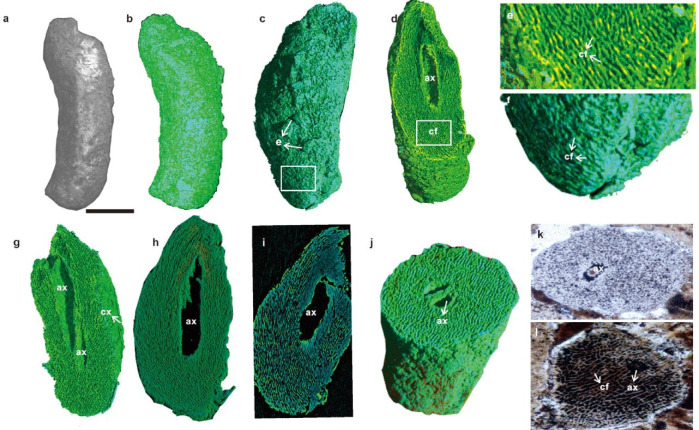
Scanning electron micrograph (SEM), SRXTM image, and thin-section images of *Vetusceramium sinense* from a phosphorite sample of WA-1008 in the Doushantuo Formation of South China. (**a**) Overview of the morphoanatomy from the SEM image. (**b**–**j**) SRXTM reconstructions of (**a**). (**b**) Overall SRXTM reconstruction. (**c**) Overall SRXTM reconstruction from the backward direction, showing several linear edges (**e**) of the potential cortex. (**d**) Half of the SRXTM reconstruction, indicating the large central axial cell (ax) and corticated cell filaments (cf). (**e**) Magnification of the rectangle in (**d**), showing the long and pericentral corticated cell filaments (cf). (**f**) Magnification of the rectangle in (**c**), showing corticated and networked cell filaments (cf) on the surface. (**g**) Half of the SRXTM reconstruction, showing two large central axial cells (ax) and a thin-layered cortex (cx). (**h**) Top part of the SRXTM reconstruction, showing the corticated cell filaments surrounding the large central axial cell (ax). (**i**) Upper part of the SRXTM reconstruction, showing fewer corticated cell filaments near the central axial cells. (**j**) Half of the SRXTM reconstruction in the cross-sectional direction, showing two axial cells divided by the cell wall. (**k**) Thin-section (WA-513) image, showing two cells divided by the cell wall. (**l**) Thin-section (WA-516) image, show cell filaments and axial cell in thallus. Scale bar, 50 μm, except for 10 μm in (**e**), (**f**), and (**m**); 100 μm in (**k**) and (**l**).

The Specimens were investigated using synchrotron radiation X-ray tomographic microscopy (SRXTM) at SPring-8 in Hyogo, Japan (23 keV, 0.388^3^ μm^3^/pixel). The detailed analytical conditions were described elsewhere^26,27^. The specimen comprises an elongated, multilayered, uniaxial thallus measuring 250 μm in length and 10–100 μm in diameter, with a basal apparatus and an apical thallus (Fig. 2a–c). Apical constriction narrows the tubular structure to 10 μm, which aligns with the early developmental branches of red algae. From a posterior perspective (Fig. 2c), cell filaments (cf) are present on the surface of the lower part (Fig. 2f). Surface linear edges (e) (Fig. 2c) may indicate growth zonation.

The basal region displays a roughened texture (Fig. 2c, f), and apical cell filaments (2 μm wide) radiate upward around a central tube (Fig. 2d, j), a morphology frequently associated with photosynthetic efficiency in extant algae. This observation supports the hypothesis that these fossil structures were capable of photosynthesis, a crucial process in the early evolution of red algae. The internal organization of the thallus, particularly the arrangement of cells and filaments, suggests a well-developed system for efficient light capture and utilization.

**Fig. 3:**
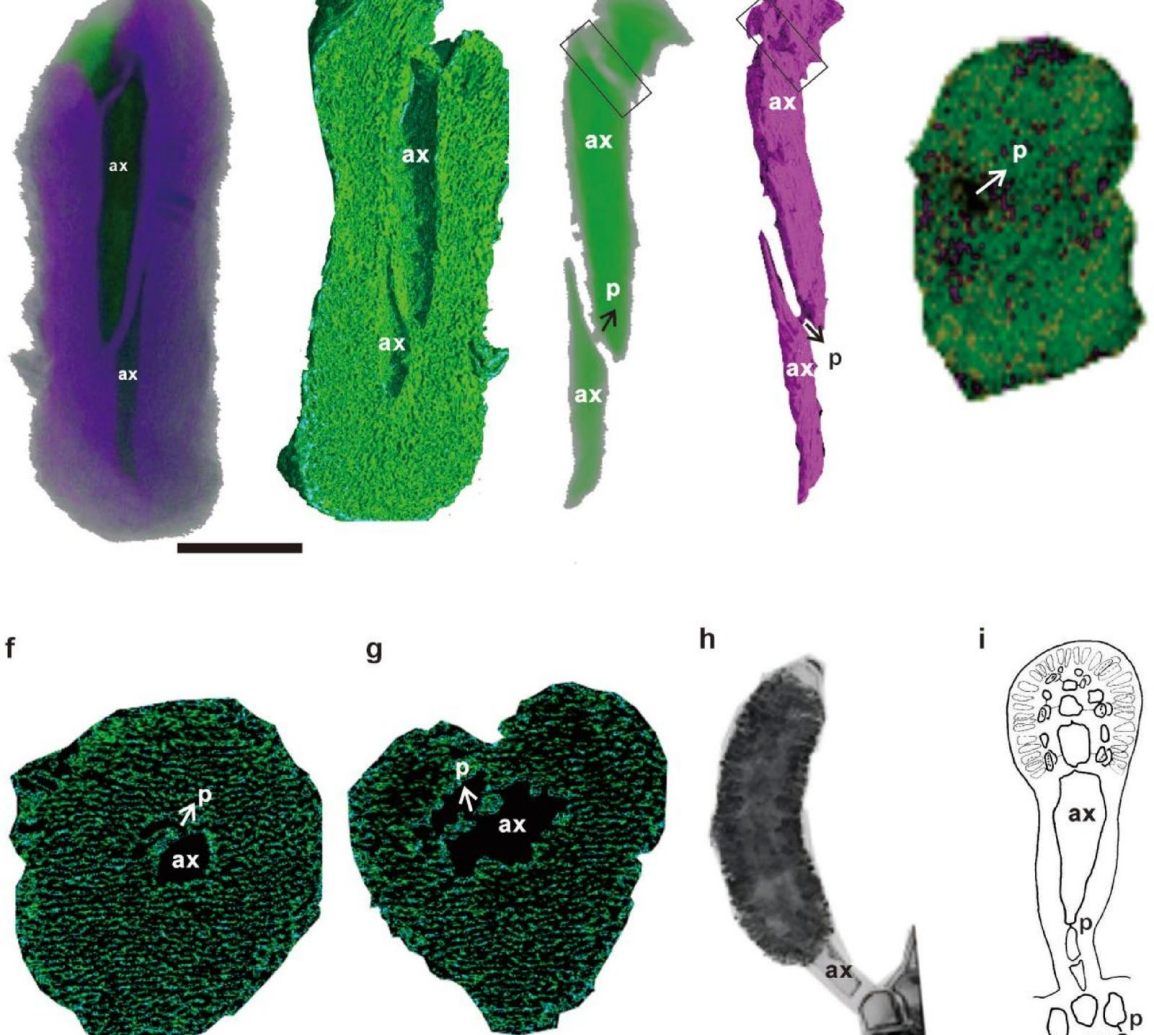
SRXTM images (**a**–**g**) of *Vetusceramium sinense* from the Doushantuo Formation of South China and images of living Ceramiales algae. (**a**) SRXTM image in the X-ray mode (Fig. 2a), revealing the distinct shape of the central axial cells. (**b**) Half of the SRXTM reconstruction, displaying central axial cells (ax) partitioned by cell walls. (**c**) and (**d**) Selected central axial cells, exhibiting pit connections (p) through the lower cell wall captured in the X-ray mode (**c**) and scatter mode (**d**). (**e**) Enlargement of the rectangle in (**c**) and (**d**), highlighting the aperture left by the pit connection. (**f**) and (**g**) Tomographic cross-sections illustrating the development of radial pit connections in the lower and upper axial cell walls. (**h**) Spermatangial branch from a living *Lophurella pseudocorticata* sp. nov.^28^ (Rhodomelaceae, Ceramiales), showing axial cells with robust septa. (**i**) Schematic drawings of spermatangial branches from modern *Pleurostichidieae*^29^ (Rhodomelaceae, Ceramiales) depicting central axial cells with pit connections. Scale bar: 50 μm in all images except for 5 μm in (**e**).

The fossil can be divided into three layers: the outermost, pericentral cell filament network, and central axial cells. The outermost layer is composed of a distinct surface thin-layered cortex (cx) (Fig. 2g), which appears to act as a protective covering for the internal structures, such as the pericentral cell filament network and central axial cells. The cells in the pericentral cell filaments are thin and corticated (Fig. 2e–i). The cell filament organization varies regionally: basal and apical filaments are thicker and parallel-aligned (Fig. 2d–f, k–j), whereas internal filaments form a radially intertwined network (Fig. 2e–f). There are fewer cell filaments near the axial cells (Fig. 2d andi). Tomographic sections (Fig. 2i) confirmed the presence of radially oriented filaments and central cylindrical axial cells (200 μm long) divided by thick septa with flaring, apical apertures (Fig. 2d and g). The axial cells taper from 25 μm (apex) to 5 μm (base) (Figs. 2g and 3a–d), and they are arranged in a single axis, which is why the thallus is uniaxial. The cross-sectional views show two axial cells divided by a cell wall (Fig. 2k) and cell filaments and axial cell in the thallus (Fig. 2l). These characteristics are common in the Weng’an thin-section samples, such as thin sections WA-513 and WA-516. The different characteristics may cause cross-sections in different directions. The SRXTM images in the X-ray mode (Fig. 3a) indicate the hold shape of the central axial cells divided by a thick cell wall. The SRXTM images of the selected axial cells exhibit pit connections (p) through the lower cell wall (Fig. 3c, d, and f). The SRXTM images of the upper axial cell wall indicate a hole left by a pit connection (Fig. 3c–e and g). The thallus is uniaxial and built around a single axial cell filament (Fig. 2 and Fig. 3).

### Interpretation

Three-dimensional reconstruction from these high-resolution images revealed an unprecedented level of detail, enabling researchers to visualize and analyze the complex internal structure of the fossilized algae (Figs. 2 and 3).

These central tube cells with pit connections may be associated with axial cells covered by a pericentral cell filament network (Fig. 2d and h), similar to those found in extant Ceramiales algae (Fig. 3h and i), which are typically spherical to cylindrical and located in the mid-region of the thalli. Beneath this surface layer, a complex network of interwoven filaments was observed (Fig. 2d and h), contributing to the overall structural integrity of the thallus. These internal filaments are arranged radially, converging towards a central axis, where they form the aforementioned axial cells. The interplay between these surface and internal features highlights the intricate architecture of the fossilized Ceramiales algae^28,29^.

The variation in cell filament organization across different regions of the fossil is particularly noteworthy. The thicker and parallel-aligned filaments in the basal and apical regions (Fig. 2d and g) likely provide additional mechanical support and stability. In contrast, the radially intertwined network of internal filaments suggests a more complex and specialized role in the physiological processes of the fossil, such as nutrient transport and cell communication.

The central axial cells, divided by thick septa and characterized by flaring apical apertures, are another key feature of the fossil. These cells likely serve as the main structural axis of the thallus, guiding the overall growth and development of the algae. The presence of pit connections between the central tube cells and axial cells further supports the notion of an efficient nutrient and water transport system within the fossil.

The fossilized tip of the algae likely represents a juvenile branch prior to thallus maturation (vegetative or tetrasporangial thallus) (Fig. 3f–j). Apical thalli with branching cell filaments are optimized for light capture in shallow aquatic habitats, supporting photoautotrophy.

The fossil exhibits a cylindrical structure with a uniaxial thallus, large central axial cell with pit connections, corticated cell filaments, and flaring apical apertures, resembling the tip of modern Ceramiales (Rhodophyta)^28,29^. The pit connections between these cells and the axial cells are also evident (Fig. 3c–g), supporting the hypothesis of an efficient internal transport system. Although the original biomolecular composition remains unresolved due to diagenetic overprinting, the specimen exhibits 3D cellular fidelity without collapse, likely preserved through the phosphatization of organic structures.

Overall, the intricate architecture and cellular organization of the fossilized Ceramiales algae provide valuable insights into the early evolution and physiological adaptations of red algae. The combination of high-resolution imaging techniques and detailed anatomical analysis has allowed for a more comprehensive understanding of these ancient organisms and their roles in the history of life on Earth.

## Discussion

Pit connections, holes in the septum between two algal cells, are unique to multicellular red algae and are crucial for understanding the early evolution of multicellular organisms and the diversification of red algal groups^15,30^. *Rafatazmia chitrakootensis*, from the ~ 1600-Ma Tirohan Dolomite of India, possesses structures comparable to pit plugs and pyrenoids^3^. Nevertheless, distinguishing putative pit plugs from diagenetic mineralization remains challenging, and there is insufficient evidence to identify pit plugs within the cells^4^. Weng’an algae^11^ were primarily identified through histology with little reference to their broader anatomy. Pit connections and pit plugs have not been observed in the fossil record^12^. Our study of *Vetusceramium sinense* provides morphological details of the pit connections. If our interpretation is correct, this would be the earliest undisputed pit connection report to date.

The order Ceramiales, the largest among red algae, comprises approximately half of the genera and one-third of the species in Rhodophyta^31^. In the normal internal branch, each axis develops from a single filament of mother cells, forming a chain of axial cells and rings of cortical cells (Fig. 2h and i). Axial cells are uniquely connected by pit connections in red algae^32^. Molecular clock estimates suggest that this group likely originated much earlier, potentially in the Paleozoic Era (*ca.* 350–400 Ma^17^), but confirming this with fossils remains difficult. Fossils definitively attributed to the order Ceramiales are exceedingly rare, and fossil evidence for the order Ceramiales remains controversial and limited. The study of these Ediacaran Doushantuo fossils, *Vetusceramium sinense,* displays a cylindrical multilayered uniaxial thallus with large central axial cells, pericentral corticated cell filaments, and possible pit connections, similar to the tip of modern Ceramiales (Rhodophyta), providing insights into the environmental conditions that favored the evolution of these algae. Furthermore, the discovery of potential Ceramiales fossils from different geological periods, such as the Eocene^16^, supports the notion of a long and complex evolutionary history of Ceramiales. These fossils offer valuable data for constructing a phylogenetic tree of Ceramiales and understanding their evolutionary relationships with other algal groups.

In conclusion, despite the limited and controversial fossil record of Ceramiales, the study of potential Ceramiales fossils from the Doushantuo Formation offers crucial insights into the early evolution and diversification of this important algal order. Future research is required to explore the morphological and molecular clock features of these fossils to further refine our understanding of the evolution of Ceramiales.

### Exclusion of green algae and metazoans

*Vetusceramium sinense* shares characteristics with the germlings of *Blidingia minima* (Ulvales green algae), including septate tubular and cylindrical thalli^33^. However, the thallus of *Blidingia minima* is soft, whereas *that of Vetusceramium sinense* exhibits 3D cellular fidelity without collapse. Additionally, the cell wall in *Blidingia minima* is thin, whereas the cell walls between the axial cells in *Vetusceramium sinense* are thick. Despite superficial similarities to the tubular, constricted thalli of the brown alga *Scytosiphon lomentaria*, the fossils lack diagnostic phaeophycean traits, such as differentiated medulla and cortex tissues^34^.

The Early Cambrian calcareous red algae of the genus *Epiphyton Bornemann* are characterized by a branching thallus^35^ and comprise spherical or rectangular capsules^36^, which resemble *Vetusceramium sinense*. However, the genus *Epiphyton Bornemann* does not exhibit regular cell filaments or central tubular structures. Moreover, *Vetusceramium sinense* does not produce calcified remains. Similarly, Ediacaran metazoans, such as *Cloudina* and *Sinotubulites,* share tubular morphologies^37^; however, the Doushantuo specimens are distinguished by their regular cell filaments, septate partitioning, and central, trumpet-shaped structures. Our fossils generally exhibit cellular preservation, which is unambiguously associated with the primary phosphatization of biological structures, rather than diagenetic artifacts.

The three-dimensionally preserved Doushantuo *Vetusceramium* from Weng’an exhibits a tube-shaped morphology similar to that of modern sponges^10,25^. However, high-resolution tomographic analysis of the walls of the specimens revealed no evidence for pores, a critical feature for filter feeding in sponges^10^. This absence indicates that *Vetusceramium* could not function as a sponge, challenging its previous morphological interpretation.

### Stem group *Ceramiales* hypothesis

Doushantuo *Vetusceramium sinense* likely represents a stem group lineage within or near Ceramiales, predating the evolutionary simplification to monolayered or bilayered thalli observed in modern taxa. The combination of large central axial cells, pericentral corticated cell filaments, pit connections, and multilayered uniaxial thallus (Fig. 3e–g), coupled with phosphatized cellular fidelity, supports their interpretation as Ceramiales algae exploring diverse body plans during the Ediacaran. However, our specimens are very limited, and the reproductive structure and overall thallus of this type of algae have not yet been observed. In the future, we will need to discover more specimens to complete the classification of algae. Despite these limitations, there are several morphological similarities between our specimens and certain species of red algae, particularly those belonging to the order Ceramiales. These similarities include branching patterns, thallus textures, and the presence of certain cellular structures. Therefore, it is plausible that *Vetusceramium* has a closer affinity with red algae than previously thought. The limitations of this study are that, while it is natural to find many 2D preserved fragments in thin sections, the number of 3D preserved specimens is limited. More 3D preserved specimens and a comprehensive collection of fossils need to be found in further studies.

## Conclusion

The exceptional three-dimensional preservation of stem group Ceramiales algae in the Ediacaran Weng’an Biota (635–551 million years ago) reveals a developmental architecture, that is strikingly similar to that of modern red algae. This discovery directly confirms that red algae with Ceramiales morphologies had already diversified in marine ecosystems over 580 million years ago, pushing back the evolutionary timeline of complex multicellular algal body plans to this period.

These fossils provide critical insights into Precambrian ecosystem composition, demonstrating that early eukaryotic life achieved sophisticated multicellularity, including large central axial cells, pericentral corticated cell filaments, pit connections, and multilayered uniaxial thallus structures. Such innovations likely underpinned the ecological success of red algae, which played a pivotal role in the Earth’s oxygenation and the establishment of benthic marine habitats prior to the Cambrian explosion.

Notably, this study marks the first report of intricate, three-dimensionally preserved multicellular algal structures resolved using synchrotron X-ray microtomography. The fidelity of the preservation of large central axial cells, pericentral corticated cell filaments, pit connections, and multilayered thalli offers an unprecedented window into the early algal evolution. These findings underscore the importance of red algae in shaping both the Earth’s biosphere and geosphere during the Neoproterozoic, bridging the gap between simple microbial communities and the rise of complex macroscopic life. This fossil also provides critical evidence for complex multicellularity in early red algae. The preservation of developmental stages offers a rare window into the life history of Ediacaran photoautotrophs.

## Systematic paleontology

Rhodophyta Wettstein, 1922.

Florideophyceae Cronquist, 1960.

Ceramiales Friedrich Oltmanns, 1904 and Taylor et al., 2008.

Ceramiaceae Dumortier, 1822.

*Vetusceramium sinense* gen. et sp. nov. (Figs. 2 and 3).

Material: Holotype WENGAN 1008.

Occurrence: Lower black bituminous facies of the Weng’an phosphorite in the Doushantuo Formation at the Weng’an section in southwest Guizhou Province, China.

Derivation of name: From the Latin “vetus,” meaning “old or ancient,” due to the arguably ancestral nature of the type compared to other modern Ceramium algae. The species was named after the discovery of fossils in China.

*Diagnosis:* The branch is an elongated cylinder, 250 μm in length and 50–100 μm in width, with large central axial cells, pericentral corticated cell filaments, pit connections, and a multilayered, uniaxial thallus.

*Description:* The specimen presents as an elongate, cylindrical thallus, measuring 250 μm in length and 10–100 μm in diameter. The fossil exhibits three distinct layers: an outermost cortex, a pericentral cell filament network, and central axial cells. The cells within the pericentral filaments are slender and corticated, whereas the central axial cells, featuring pit-connections traversing thick cell walls, preserve their morphological integrity. The thallus is uniaxial, constructed around a solitary axial cell filament.

## Supplementary Information

Below is the link to the electronic supplementary material.

Supplementary Material 1

Supplementary Material 2

Supplementary Material 3

Supplementary Material 4

## Data Availability

All data supporting the findings of this study are available within the paper and its Supplementary Information. The raw data are provided in Supplementary Video 1. All data generated or analyzed during this study are included in this published article [and its supplementary information files].
