# Supplementary material for: Three-dimensional reconstruction of ediacaran ceramiales (Rhodophyta) from the phosphorite doushantuo formation, South China
Source: Sci Rep. 2026 Mar 6;16:9935. doi: 10.1038/s41598-026-42410-5 (PMC13021990; doi:10.1038/s41598-026-42410-5)
Supplement: Supplementary file 4 — Supplementary Material 4 [file 41598_2026_42410_MOESM4_ESM.docx]

## Additional information

**Supplementary information includes three movies:**

Captions for movies 1-3:

Movie 1: Reconstructed slice data for these specimens (Fig. 2), on which the 3D rendered models were created. These files are downsampled versions of the original files to provide easy access to the data.

Movie 2: 3D image movie of the fossil reconstructed from the SXRTM images captured in the X-ray mode (Fig. 2a), revealing the distinct shapes of the central axial cells.

Movie 3: 3D image movie of selected central axial cells in scatter mode.
